# Self-Nanoemulsifying Drug Delivery Systems for Enhancing Solubility, Permeability, and Bioavailability of Sesamin

**DOI:** 10.3390/molecules25143119

**Published:** 2020-07-08

**Authors:** Chih-Yuan Wang, Ching-Chi Yen, Mei-Chich Hsu, Yu-Tse Wu

**Affiliations:** 1 School of Pharmacy, College of Pharmacy, Kaohsiung Medical University, Kaohsiung 80708, Taiwan; s6510624@gmail.com (C.-Y.W.); date0315@hotmail.com (C.-C.Y.); 2 Department of Sports Medicine, Kaohsiung Medical University, Kaohsiung 80708, Taiwan; 3 Department of Medical Research, Kaohsiung Medical University Hospital, Kaohsiung 80708, Taiwan; 4 Substance and Behavior Addiction Research Center, Kaohsiung Medical University, Kaohsiung 80708, Taiwan; 5 Drug Development and Value Creation Research Center, Kaohsiung Medical University, Kaohsiung 80708, Taiwan

**Keywords:** sesamin, self-nanoemulsifying drug delivery system, oral bioavailability

## Abstract

Sesamin (SSM) is a water-insoluble compound that is easily eliminated by liver metabolism. To improve the solubility and bioavailability of SSM, this study developed and characterized a self-nanoemulsifying drug delivery system (SNEDDS) for the oral delivery of SSM and conducted pharmacokinetic assessments. Oil and surfactant materials suitable for SNEDDS preparation were selected on the basis of their saturation solubility at 37 ± 0.5 °C. The mixing ratios of excipients were determined on the basis of their dispersibility, transmittance (%), droplet sizes, and polydispersity index. An SNEDDS (F10) formulation comprising glyceryl trioctanoate, polyoxyethylene castor oil, and Tween 20 at a ratio of 10:10:80 (*w*/*w*/*w*) was the optimal formulation. This formulation maintained over 90% of its contents in different storage environments for 12 weeks. After the self-emulsification of SNEDDS, the SSM dispersed droplet size was 66.4 ± 31.4 nm, intestinal permeability increased by more than three-fold, relative bioavailability increased by approximately 12.9-fold, and absolute bioavailability increased from 0.3% to 4.4%. Accordingly, the developed SNEDDS formulation can preserve SSM’s solubility, permeability, and bioavailability. Therefore, this SNEDDS formulation has great potential for the oral administration of SSM, which can enhance its pharmacological application value.

## 1. Introduction

Sesame (*Sesamum indicum* L.) is part of the Pedaliaceae family, and it is planted in areas ranging from tropical to temperate zones in Africa, Asia, and Latin America. The history of sesame consumption can be traced back to Assyria and Babylon 4000 years ago [1]. Sesame has several nutritional and health benefits. Studies on senescence-accelerated mice have revealed that the levels of reactive carbonyl species (RCs) in the cerebral cortex and liver increase with age. Basal diet supplementation with sesame lignan for 10 months was reported to reduce the levels of RCs in mice. Research has also revealed that sesame lignans suppress cognitive decline by reducing age-related RCs production in the brain [2]. In addition, a study reported that the treatment of ketoconazole-induced liver injury in mice through the oral administration of sesame oil (0.5 mL/kg) for 14 days substantially reduced concentrations of serum aspartate aminotransferase and alanine aminotransferase; no statistically significant differences were observed between the mice with injury and the healthy group. This indicates that sesame oil regulates the redox system of the liver and has antioxidant activity [3]. Another study demonstrated that sesame lignan (50 mg/kg) consumption by rabbits for 4 weeks significantly reduced the levels of proinflammatory cytokines and macrophage infiltration in the liver. Moreover, sesame lignans were found to regulate the plasma platelet-activating factor acetylhydrolase and eliminate prolongation of the low-density lipoprotein (LDL) oxidation lag time [4]. The findings of the aforementioned studies indicate that sesame has considerable physical benefits in regulating aging factors, liver function, and LDL.

Sesamin (SSM), a lignan abundantly present in sesame seeds, exhibits various physiological effects. A recent study demonstrated its anti-inflammatory effects in a rat paw inflammation model; specifically, the study revealed that the oral administration of SSM at a dose of 50–200 mg/kg reduced swelling, and the anti-inflammatory effects increased with the SSM dose [5]. Another study indicated that SSM administration could extend the lifespan of *Caenorhabditis elegans* through dietary restriction-related signaling pathways [6]. In addition, a study confirmed that SSM administration (0.35 and 2 mg/mL) in a *Drosophila* senescence-accelerated model upregulated four antioxidant and two DNA repair genes simultaneously. Accordingly, SSM can significantly improve the lifespan of flies. SSM also plays a major role in antifatigue effects [7,8] and immunity regulation [9]. SSM supplementation may become essential for people who value long-term care and health supplementation.

Although several studies have demonstrated the advantages of SSM, the poor solubility of SSM in water (2.5 μg/mL) has considerably limited the dissolution rates of its components and its release efficiency [10]. Moreover, the poor aqueous solubility of SSM has prevented a discussion of its class in the biopharmaceutics classification system (BCS). However, considering the numerous benefits of SSM, investigating its properties further would be of high value. A previous study by Tomimori et al. [11] applied [^14^C] sesamin to assess the pharmacokinetics and mass balance of sesamin in rats. The results indicated that plasma SSM was detected in trace amounts, and the radioactivity of SSM was about 1% of that of the major conjugated metabolite (SC-2) after oral administration, which suggested the extensive first-pass metabolism of SSM. To enhance the absorption and distribution of SSM, a study proposed an approach that entails preparing a dosage formulation by dissolving sesame extract in turmeric oil and homogenizing it after emulsification [12]. The median droplet size of the dosage formulation obtained was approximately 0.58 μm, which is larger than that from common microemulsion, and it may have limited effects on passive diffusion in the gastrointestinal tract. Nevertheless, this study only discussed the drug distribution and dose-dependent contents of sesame extract in serum and the brain and lacked pharmacokinetic data. Furthermore, it has been reported that solid dispersion can increase the solubility and bioavailability of SSM [10]. Although components existing in an amorphous state in solid dispersion can improve the solubility, such a formulation has the disadvantage of being unstable. The amorphous components may recrystallize and reduce drug release during the preparation or storage stages (especially in environments with a high temperature and humidity) [13]. Moreover, the study involved a 12-h pharmacokinetic test and a 4-week storage stability test. Therefore, its results cannot provide sufficient pharmacokinetic and storage stability parameters for discussion.

To improve the absorption and distribution of lipophilic components, various nanocarriers have been reported to increase the characterization of such components by enhancing their solubility and permeability. Among such nanocarriers, lipid-based drug delivery systems, such as self-nano-emulsifying drug delivery systems (SNEDDSs), have been extensively used for increasing the oral bioavailability of pharmaceutical components [14]. An SNEDDS is an isotropic mixture of an active pharmaceutical ingredient, oil, surfactant, and cosurfactant. Its components are uniformly dispersed in the self-emulsifying matrix, and it has a 100% drug entrapment efficiency [15]. Following dispersion in an aqueous environment through gentle agitation, the SNEDDS will spontaneously emulsify and form o/w droplets measuring 20–200 nm in size [16]. In addition to enhancing the solvent capacity and scalability, an SNEDDS enhances drug permeation across the intestinal membrane and diminishes food effects on drugs.

The aim of the present study was to develop an optimized SSM-loaded SNEDDS formulation to substantially enhance the solubility and bioavailability of SSM. We selected the optimal SSM–SNEDDS formulation by verifying the droplet size, transmittance (%), and dispersibility. Subsequently, we evaluated its effect on the intestinal permeability and storage stability under accelerated conditions. We used rat models to study the pharmacokinetic behavior of SSM in order to clarify possible means of enhancing its absorption.

## 2. Results and Discussion

### 2.1. SNEDDS Characterization and Optimization

#### 2.1.1. SSM Solubility Assessment

**Figure 1:**
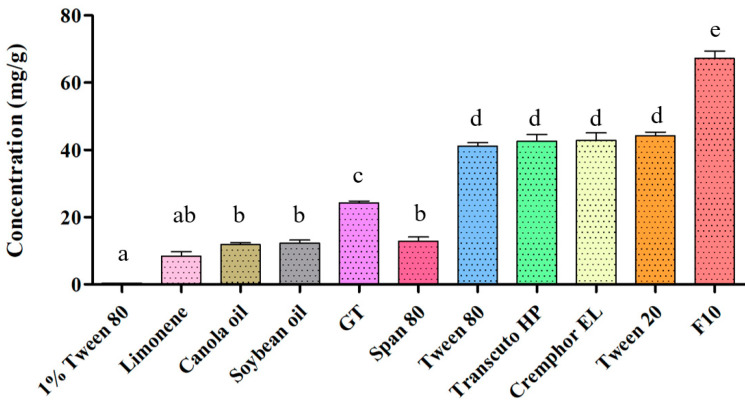
The solubility of sesamin (SSM) in different solvents. Results are expressed as the mean ± standard error of the mean (*n* = 3 for each group). GT is glyceryl trioctanoate, F10 is the composition of GT:Cremophor EL:Tween 20 = 1:1:8 (*w*/*w*/*w).* Values that do not share the same letter are significantly different (*p* < 0.05).

First, a solubility experiment was conducted to characterize and optimize the SNEDDS, and Figure 1 presents the results. The results revealed that among all oil phase materials, the solubility of SSM was the highest in glyceryl trioctanoate (GT; 24.2 ± 0.4 mg/g). In addition to its solubility in sorbitan monooleate (Span 80), the solubility levels of SSM in the surfactants and cosurfactants Tween 20, Tween 80, polyoxyethylene castor oil (Cremophor EL), and Transcuto HP did not differ significantly. The SSM solubility in the optimized formulation F10 was 67.3 ± 2.2 mg/g at 37 ± 0.5 °C. Compared with the SSM solubility in 1% Tween 80 aqueous solution, the solubility in F10 was significantly improved by approximately 160 times.

Soybean oil and canola oil are composed of linoleic acid with different chain lengths (C16–C18), mixed with different proportions of saturated and unsaturated fatty acids [17]. Limonene is a terpene with a monoterpene structure. GT is a triglyceride with three medium-chain fatty acids (C8). This explains why the solubility of SSM was significantly higher in GT than in soybean oil, canola oil, and limonene. Furthermore, GT is a medium-chain triglyceride (MCT), whose absorption does not involve chylomicron production. The small intestine has a better capacity to absorb MCTs than it does long-chain triglycerides because it can digest MCTs entirely [18]. Accordingly, we considered GT the most suitable oil phase for synthesizing the SSM–SNEDDS combination.

#### 2.1.2. Dispersibility and Transmittance (%) of the SNEDDS Composition

Formulations prepared using different ratios of oils and surfactants were evaluated and screened through droplet size analysis, a transmittance test, and a dispersibility test to determine the optimal formulation, and Table 1 lists the results. The results indicated that F1–F5 were poorly dispersed, exhibiting turbidity after dispersion. Moreover, F6–F9 exhibited a slightly milky white appearance after dispersion, and the corresponding transmittance was less than 40%. F10–F13 exhibited a clear and transparent appearance, and their transmittance levels were similar (>90%). As shown in Table 1, the raised percentages of Transcutol HP in formulations (F6–F9) resulted in an increase of the droplet size. This phenomenon was caused by the inflow of a large number of Transcutol HP molecules into the oil layer, which led to interfacial film expansion and particle size accretion. As a result, the emulsion gradually turned cloudy and turbid [19]. Cremophor and Tween are two types of nonionic surfactants, which are commonly used for the development of nanoemulsions. Previous studies have revealed that the molecular features of emulsifiers can affects the formation of self-emulsifying formulations [20]. The molecular features of an emulsifier can be characterized by the packing parameter (PP), which can influence the curvature of the monolayer between the oil-water interface [21]. The molecular features of Tween notably included a large head group and a rather short tail, and the PP of Tween was estimated to be 0.07 [22]. Compared with Tween, Cremophor EL possesses a stronger emulsifying capacity because of its larger amount of ethylene oxide, and Cremophor EL is more compatible with the oil phase [23]. In addition, the hydrophilic groups (polyethylene glycols and ethoxylated glycerol) of Cremophor EL could be incorporated with the aqueous phase, and the viscosity diversity between the oil-water interface was minimized, which caused a reduction in the critical Weber number and an increment of the droplet break-up efficiency [24]. Another study concluded that the PP of Cremophor is closer to the intermediate value than Tween, because the intermediate PP is crucial for the emulsifier to form fine oil droplets and transparent nanoemulsions [20].

**Figure 2:**
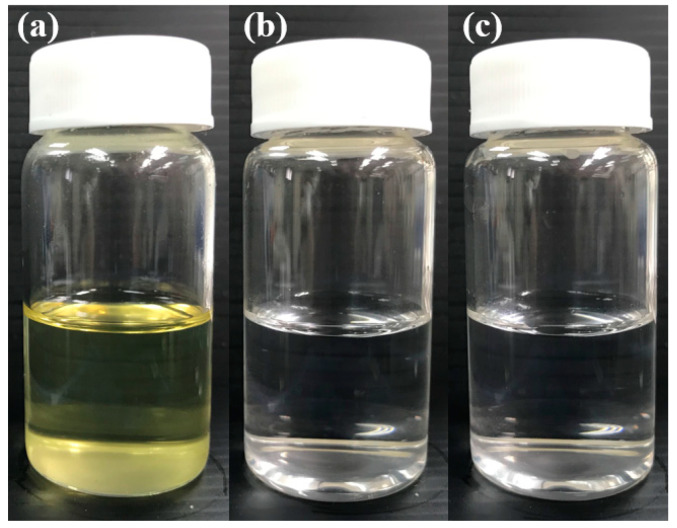
(**a**) SSM–SNEDDS appearance; SSM–SNEDDS dispersed in: (**b**) water; and (**c**) 0.1 N HCl at a ratio of 1:100 (*v*/*v*).

After a comprehensive comparison, we selected the F10 formulation as the optimal combination; its droplet size and polydispersity index (PDI) values were 66.4 ± 31.4 nm and 0.05 ± 0.01, respectively. F10 exhibited a favorable dispersibility and the potential to increase the absorption and distribution. A favorable dispersibility is critical to the achievement of an excellent SNEDDS composition. We visually assessed the dispersibility of the different SNEDDS formulations in vitro by using a previously described grading system [25]. In this grading system, a formulation with an evenly dispersed emulsion with a clear or slightly bluish appearance is classified as grade A; a formulation with poor emulsification and large oil droplets on the surface is classified as grade E. Our results revealed that F10–F13 were rapidly emulsified and dispersed in water or 0.1 N HCl. Therefore, they were classified as grade A (Figure 2). The other formulations required a longer emulsification time; occasionally, they did not undergo emulsification reactions. After comparing the observed dispersibility, light transmittance, particle size, and PDI of the formulations, we selected F10, exhibiting an average droplet size of <100 nm, as the optimal formulation.

### 2.2. Droplet Sizing and Morphological Characterization

**Figure 3:**
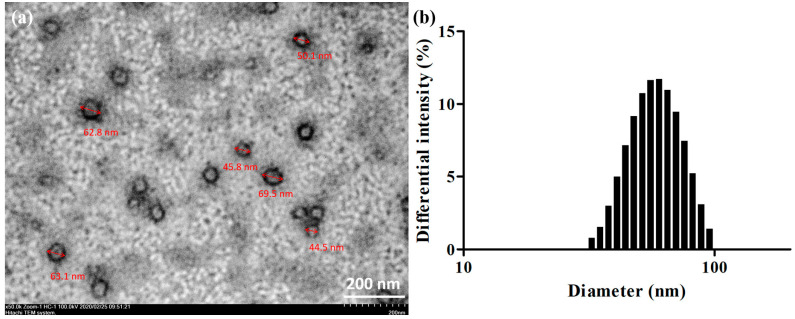
Characterization of F10 morphological observation and droplet sizing: (**a**) The transmission electron microscopy micrographs and (**b**) droplet size distribution.

Figure 3b illustrates the particle size analysis results. F10 comprised droplets measuring approximately 66.4 ± 31.4 nm. The droplets were round and well dispersed when observed through transmission electron microscopy (TEM).

### 2.3. Stability Study

**Figure 4:**
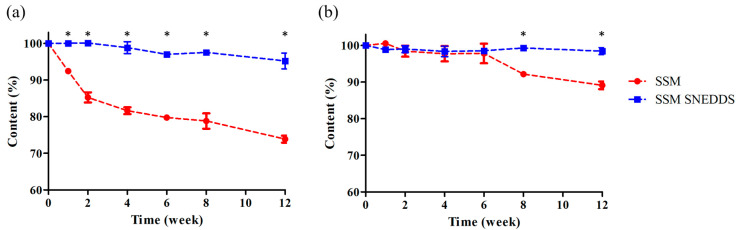
Contents stability of SSM powder and SSM–SNEDDS in the (**a**) acceleration and (**b**) long-term stability examination for 12 weeks. Results are expressed as the mean ± standard deviation of the mean (*n* = 3 for each group). * *p* < 0.001, compared with the SSM group.

An accelerated stability test was conducted for SSM powder and the SSM–SNEDDS formulation at a constant temperature and relative humidity of 40 ± 2 °C and 75 ± 5%, respectively; the test lasted 12 weeks. The results indicated that after the first week, the content of the SSM powder decreased to approximately 90%. After 12 weeks, the contents of the SSM powder and SSM–SNEDDS were 73.9 ± 1.0% and 95.2 ± 2.2%, respectively. Furthermore, a long-term stability test was conducted at a constant temperature and humidity of 25 ± 2 °C and 60 ± 5%, respectively; the test also lasted 12 weeks. The results revealed that the contents of the SSM powder and SSM–SNEDDS were 89.1 ± 1.0% and 98.4 ± 1.1%, respectively (Figure 4). After 12 weeks of storage, the content of SSM–SNEDDS was more than 95%. These results suggested that the encapsulation of SSM in the SNEDDS may provide a sufficient stability for at least 12 weeks. Accordingly, under the same storage conditions, SSM–SNEDDS would exhibit a superior stability in comparison to SSM powder.

### 2.4. Permeability Evaluation

**Figure 5:**
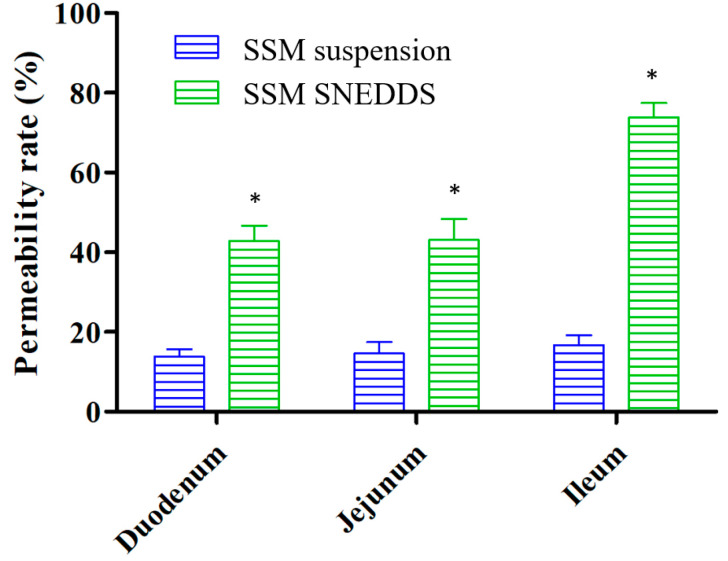
Permeability evaluation of SSM suspension and SSM–SNEDDS in different small intestine segments. Different small intestine segments were incubated in Tyrode’s solution containing SSM suspension or SSM–SNEDDS at 37 ± 0.5 °C for 1 h. Results are expressed as the mean ± standard deviation of the mean (*n* = 6 for each group). * *p* < 0.001, compared with the group of SSM suspension.

An intestinal permeability experiment was conducted for SSM. The experimental results (Figure 5) indicated that the permeability of SSM encapsulated in the SNEDDS dosage form exhibited a trend of improvement for the various intestinal segments. Specifically, the SSM permeability increased from 13.8 ± 1.9% to 42.8 ± 3.9% in the duodenum, from 14.6 ± 2.9% to 43.1 ± 5.2% in the jejunum, and from 16.7 ± 2.5% to 73.8 ± 3.7% in the ileum. The permeability of SSM thus increased by 3- to 4.4-fold in each intestinal segment, representing a statistically significant enhancement (*p* < 0.05).

The solubility and permeability of drugs are usually determined in accordance with the BCS specifications. The solubility of SSM in water is approximately 2.5 μg/mL [10]. A previous study reported that compounds with a poor water solubility (<100 μg/mL) are mostly classified as belonging to BCS class II [26]. BCS class II compounds have a low solubility and high permeability; thus, solubility enhancement is the determining step for improving the physical properties of such compounds. Studies have yet to determine the BCS class for SSM. In the present study, we observed that an increase in the solubility of SSM enhanced its permeability. Therefore, we can speculate that SSM belongs to BCS class II.

### 2.5. Pharmacokinetic Study

**Figure 6:**
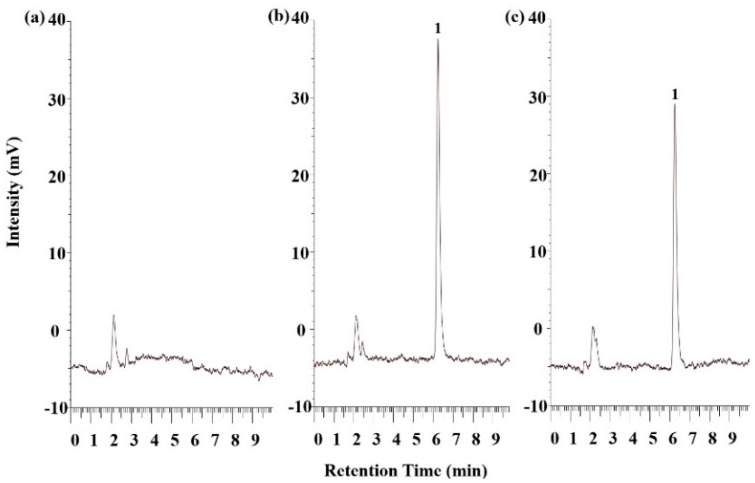
The HPLC-fluorescence chromatograms of (**a**) blank rat plasma; (**b**) blank rat plasma spiked with SSM (20 ng/mL); and (**c**) a rat plasma sample at 5 min after the oral administration of F10 (100 mg/kg). Peak identification: (1) SSM.

Figure 6 displays the chromatograms of rat blank plasma, rat blank plasma spiked with standard SSM, and a plasma sample obtained 5 min after the oral administration of 100 mg/kg SSM–SNEDDS. We confirmed that the peak of SSM was free from endogenous interference or metabolites by comparing the chromatogram of blank rat plasma (Figure 6a) with that of the blank rat plasma spiked with SSM (Figure 6b). The chromatograms demonstrated the good specificity of the analytical method and the reliability of acquired data. A previous study showed that daily SSM treatment (80 and 160 mg/kg) for 16 weeks significantly improved left ventricular hypertrophy and fibrosis in spontaneously hypertensive rats [27]. In addition, an 8-week oral administration of SSM (100 mg/kg per day) significantly improved the pathological lesions of liver fibrosis in rats induced by CCl_4_ [28]. Another study demonstrated that a 4-week oral administration of SSM (100 mg/kg per day) can significantly modulate the heart rate and blood pressure in streptozotocin induced diabetes rats, suggesting the potential cardioprotective effects of SSM [29]. Therefore, we chose a dose of 100 mg/kg for the pharmacokinetic evaluation of SSM.

**Figure 7:**
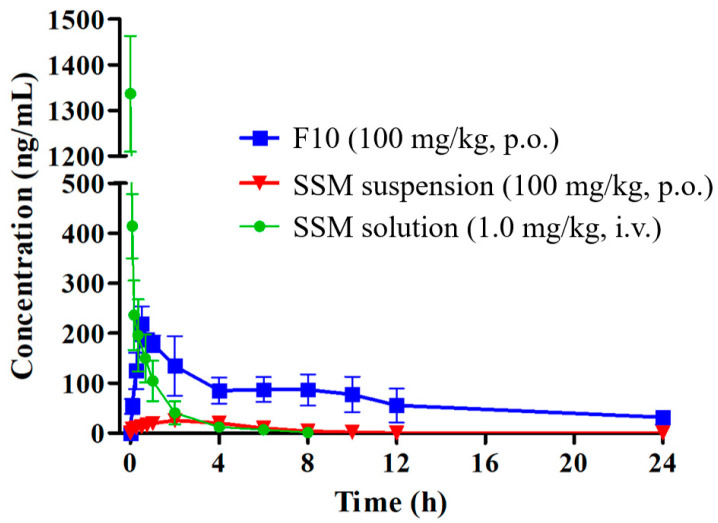
The plasma concentration–time profiles of SSM after drug administration in rats: (■) indicates F10 (100 mg/kg, p.o.); (▼) indicates SSM suspension (100 mg/kg, p.o.); (●) indicates SSM solution (1 mg/kg, i.v.); data are expressed as the mean ± standard deviation of the mean (*n* = 6 for each group).

Figure 7 presents blood concentration–time curves for each group of rats, and Table 2 lists the pharmacokinetic parameters. The rats were orally (p.o.) administered the optimized SSM–SNEDDS (F10) and SSM suspension (suspended in 2%, *w*/*v*, carboxymethyl cellulose) at a dose of 100 mg/kg and were intravenously (i.v.) administered SSM (1 mg/kg) dissolved in a mixture of dimethyl sulfoxide (DMSO) and PEG 400 (50:50, *v*/*v*). The pharmacokinetic parameters of SSM improved significantly with the SSM–SNEDDS formulation. Maximum plasma concentrations (C_max_) of 25.6 ± 3.9 and 231.2 ± 15.3 ng/mL were observed for SSM in SSM suspension and SSM–SNEDDS, respectively. Therefore, C_max_ showed a nine-fold increase. The area under the curve observed for SSM in SSM–SNEDDS (1697.9 ± 624.7 h·ng/mL) was greater than that observed for SSM in SSM suspension (131.9 ± 26.0 h·ng/mL). The relative bioavailability of SSM in the SSM–SNEDDS formulation increased by approximately 12.9-fold compared with that in the SSM suspension formulation. Additionally, the results obtained after intravenous (IV) bolus administration indicated that the absolute bioavailability rates observed for SSM–SNEDDS and SSM suspension were 4.4% and 0.3%, respectively. A previous work has reported the application of α-glycosylated stevia as the excipient for preparing solid dispersion SSM by the spray-drying method [10]. The results indicated that this solid dispersion formulation provided a C_max_ of 793 ng/mL, the time to maximum concentration (T_max_) of 8 h, and a 30-fold improvement of the oral bioavailability of SSM when compared with that of the crystalline one. In addition, the study by Tomimori et al. [11] indicated that the total radioactivity recovery in urine and bile was approximately 94%, suggesting a high oral absorption of SSM. The pharmacokinetic results revealed that the C_max_ was 2190 ng*eq./mL, and the bioavailability was 54.1%. However, the radioactivity of SSM was only 1% of that of one major conjugated metabolite (SC-2) after oral administration, and unchanged SSM was not detected in feces. These results indicated that SSM underwent extensive first-pass metabolism, and SSM mainly existed as conjugated forms in plasma. The extremely low oral bioavailability of SSM can primarily be attributed to its poor aqueous solubility and extensive first-pass effect in the liver [11]. Therefore, we used the SNEDDS formulation to improve the absorption of SSM. As a result that SSM is lipophilic, its intestinal absorption is affected by bile production and a high-fat diet [30]. F10 in this study was composed of GT, cosurfactants, and surfactants (10:10:80, *w*/*w*/*w*), with only 10% oil phase materials (*w*/*w*). As a lipid-based drug formulation, SNEDDS has advantages such as thermodynamic stability, shelf stability, and high drug entrapment [14]. Triglycerides were also part of the composition of SNEDDS because they could easily form chylomicrons and pass through the lymphatic system [31].

Upon oral administration, the SNEDDS undergoes a self-emulsifying process in gastrointestinal fluids and forms o/w micelle droplets with a size of <100 nm [32], which are easy to disperse and promote a component distribution. The components exist in the interior of the droplets, thus protecting them from acidic hydrolysis in the gastrointestinal tract and eliminating the effects of food on the drug. [14]. Moreover, nanometer-sized droplets play a key role in the absorption and distribution of components. The encapsulation of SSM in the oil-in-water droplet may prevent SSM from possible pre-systemic degradation caused by digestive fluid, enzymes, and microflora in the intestinal tract [33]. In addition, the nanoemulsion may mimic chylomicrons, and can thus improve the intestinal absorption and lymphatic transport of poorly water-soluble compounds [34].

## 3. Materials and Methods

### 3.1. Chemicals and Reagents

Sesame extract (SSM > 90%) was obtained from Bio-true (Taichung, Taiwan). Cremophor EL and GT were supplied by Sigma-Aldrich (St Louis, MO, USA). Diethylene glycol monoethyl ether (Transcutol HP) was supplied by Gattefossé (Cedex, Saint-Priest, France). (+)-Limonene was purchased from Acros Organics (Morris, NJ, USA). Polysorbate 80 (Tween80), polysorbate 20 (Tween20), Span 80, acetonitrile for high-performance liquid chromatography (HPLC), and SSM standard (purity > 98.0%) were obtained from Tokyo Chemical Industry (TCI, Tokyo, Japan).

### 3.2. Solubility Study

Before SNEDDS dosage formulation preparation, the excipient materials were screened. The oil phase materials constituting the formulations were limonene, GT, canola oil, and soybean oil, and the surfactants were Span80, Cremophor EL, Tween 80, Tween 20, Transcuto HP, and Tween 80 solutions (1%, *w*/*v*). The solubility test was conducted using a method provided by a previous study [35]. SSM was added to the excipients and then mixed using a vortex mixer for 30 s; subsequently, the mixture was placed in a thermostatic water-shaking bath and shaken at 50 rpm for 48 h at 37 ± 0.5 °C to determine saturation equilibrium. Next, the samples were centrifuged at 9400× *g* for 15 min, after which 100 μL of each sample’s supernatant was diluted with an appropriate acetonitrile solution. Finally, the SSM content of each excipient was quantified through reverse HPLC–ultraviolet (UV) analysis.

The HPLC system (Hitachi, Tokyo, Japan) employed in this study comprised an L-2130 pump, L-2200 autosampler, and L-2420 UV–visible (Vis) detector. SSM excipients were separated using a reversed-phase Luna C18 column (250 × 4.6 mm, 5 μm; Phenomenex, Torrance, CA, USA). The mobile phase consisted of acetonitrile and distilled water at a ratio of 70:30 (*v*/*v*), and its flow rate was 1.0 mL/min. The sample injection volume and UV detection wavelength were 20 μL and 290 nm, respectively. The assay conducted for the SSM concentration exhibited a linear range of 0.5–100 μg/mL (R^2^ = 0.9997, limit of quantification, LOQ = 0.5 μg/mL).

### 3.3. SSM SNEDDS Preparation

The SSM–SNEDDS preparation procedure was as follows. First, GT (oil phase) and Tween 20 (surfactant) were evaluated and compared at ratios of 1:1, 1:3, 1:5, 1:7, and 1:9 (*w*/*w*), indicating that the GT was maintained at 1 g. After characterization results were validated, the oil–surfactant ratio was maintained at 1:9. Cremophor EL and Transcuto HP were then used as cosurfactants to adjust and prepare the SNEDDS at oil–co-surfactant–surfactant ratios of 1:1:8, 1:2:7, 1:3:6, and 1:4:5 (*w*/*w*/*w*); each group’s batch size was maintained at 10 g. The droplet size, transmittance (%), and dispersibility of the formulations were compared. After a thorough analysis, we selected the optimal formulation to be F10 (GT–Cremophor EL–Tween 20 ratio = 1:1:8, *w*/*w*/*w*). The F10 preparation procedures were as follows: First, GT was weighed in a sample vial, and Cremophor EL and Tween 20 were subsequently added dropwise to the quantitative volume. The mixture was then shaken until it was evenly mixed. Finally, SSM was added to the SNEDDS dosage formulation. The vial was submerged in an ultrasonic water bath for 30 min to completely dissolve the SSM powder. The prepared SSM–SNEDDS was stored in a sealed glass bottle at room temperature until use.

### 3.4. Dispersibility Test and Percentage Transmittance

A dispersibility test and transmittance test were conducted to assess the degree of SNEDDS emulsification. In the dispersibility test, 1 mL of each SNEDDS sample was dropped into 500 mL of purified water or 0.1 N HCl at 37 ± 0.5 °C with paddle rotation at 50 rpm in an SR8 PLUS dissolution apparatus (Hanson Research, Chatsworth, CA, USA); the apparatus provided gentle agitation to promote self-emulsification. SNEDDS dispersibility was graded using a system presented by a previous study [25]. To measure the percentage of transmittance, all SNEDDS formulations were dissolved in water and diluted 100 times. Purified water was taken as a blank control and analyzed using a UV–Vis spectrophotometer (U-5100, Hitachi, Tokyo, Japan).

### 3.5. Morphological Characterization and Droplet Sizing

#### 3.5.1. Droplet Size and Distribution Analysis

To analyze the emulsified droplet size of each SNEDDS formulation, each formulation was diluted 500 times with Mini-Q water (0.2%, *w*/*v*), yielding a clear or weak opalescent dispersion. Subsequently, 3 mL of solution was collected and filtered using a 0.45-μm filter. The sample particle size was assessed using an ELSZ-2000 particle size analyzer (Otsuka Electronics, Otsuka, Japan). Measurements were performed in triplicate. From the analysis, we obtained the droplet size (mean ± standard deviation), PDI, and size distribution by the volume intensity.

#### 3.5.2. TEM

The droplets of the optimized SSM–SNEDDS formulation were examined through TEM. Specifically, SSM–SNEDDS was diluted 400 times with Mini-Q water (0.5%, *w*/*v*) and dropped on a 300-mesh copper grid coated with carbon. After negative staining with 2% (*w*/*v*) phosphotungstic acid (PTA) solution, excess PTA was removed using Mini-Q water. The copper grid was then placed in a vacuum drying oven for 2 days. The dried grid was observed through TEM using an HT7700 instrument (Hitachi, Tokyo, Japan) at a voltage of 100 kV. The ImageJ (National Institutes of Health, Bethesda, MD, USA) was used to confirm the droplet size.

### 3.6. Stability Study

To investigate the stability of SSM in the powder state and SSM contents in the SNEDDS formulation, the SSM powder and SSM–SNEDDS (10 mg/g) (*n* = 3) were packed into sample vials individually, sealed, and placed in a chamber with a constant temperature and humidity until sampling and analysis. Long-term and accelerated stability tests were conducted for the SSM–SNEDDS combination over a period of 12 weeks. For the long-term stability test, the temperature and relative humidity were maintained at 25 ± 2 °C and 60 ± 5%, respectively; for the accelerated stability test, the temperature and humidity were maintained at 40 ± 2 °C and 75 ± 5%, respectively.

### 3.7. Experimental Animal Preparation

Male Sprague-Dawley rats (weight of 200 ± 20 g) purchased from the BioLASCO Experimental Animal Center (Taipei, Taiwan) were used in this study. The animal experimental protocols were reviewed and approved by the Institutional Animal Care and Use Committee (No. IACUC-108052) of Kaohsiung Medical University Hospital (Kaohsiung, Taiwan). The rats were bred in cages with sufficient food and water (3 rats/cage) until 12 h before the SSM pharmacokinetic experiments. Their feeding environment had a 20 ± 1 °C temperature, 60 ± 10% relative humidity, and 12-h light/dark cycle.

### 3.8. Permeability Evaluation

Before observing SSM’s permeability, SSM was added to 2% carboxymethyl cellulose (CMC) colloidal solution (*w*/*v*) to prepare the SSM suspension. The SSM SNEDDS and SSM suspension were then dispersed in Tyrode’s solution, and the obtained SSM concentration was 50 µg/mL. The permeability evaluation was conducted using a method described in a previous study [35]. In brief, after each rat was anesthetized, its gastrointestinal tract was removed, and the entire small intestine was isolated. The intestine was separated into three segments according to a previous study [36]: the duodenum, jejunum, and ileum. The segments were flushed with normal saline (0.9%, *w*/*v*) and placed in a fresh sample of Tyrode’s solution. Each intestinal segment was cut into a section measuring approximately 4 cm, turned inside out, and tied at one end. After being filled with 300 μL of Tyrode’s solution, the open ends of the intestinal segments were ligated to form small sacs. Each section of the small intestinal sac was placed in Tyrode’s solution containing SSM suspension or SSM–SNEDDS (50 µg/mL). The samples were loaded in a centrifuge tube and shaken in a constant-temperature water bath (50 rpm, 37 ± 0.5 °C) for 1 h. Finally, the solution in the intestinal sac was removed, and SSM was extracted using a liquid–liquid extraction method. The SSM contents of each sample were analyzed using the HPLC–UV method.

### 3.9. Pharmacokinetic Study

The pharmacokinetic study was performed using a parallel design. A total of 18 Sprague-Dawley rats (weight of 240 ± 10 g) were randomly assigned to three groups, with each group having six rats. The rats were subjected to overnight fasting for 12 h and were administered the optimized SSM SNEDDS formulation or SSM suspension orally (100 mg/kg, p.o.). The orally administered SSM suspension was composed of SSM and 2% CMC solution (*w*/*v*). For repetitive blood sampling, we applied a jugular vein catheterization model [37]. The rats were anesthetized, and polyethylene tubes were implanted into the right jugular veins. Blood samples (300 μL) were collected in heparinized tubes before dosing (0 min); at 5, 15, and 30 min after dosing; and at 1, 2, 4, 6, 8, 10, 12, and 24 h after dosing. For the IV bolus administration group, each rat was administered the SSM solution formulation (1 mg/kg) through the femoral vein; the IV solution comprised DMSO/PEG 400 (50:50, *v*/*v*). Blood samples were collected as blanks before IV administration; at 1, 5, 10, 20, and 40 min after IV administration; and at 1, 2, 4, 6, and 8 h after IV administration. An equal volume of heparinized normal saline replaced the blood removed at each sampling point. Each sample was centrifuged at 3000× *g* for 10 min, and the plasma supernatant was separated and stored at −70 °C for analysis within 24 h.

### 3.10. Plasma Sample Preparation and HPLC Validation

To analyze the SSM content in plasma, extraction was performed first. The extraction method applied in this study was adapted from a previous study [37]. First, 100 μL of each plasma sample was added to 500 μL of ethyl acetate and then shaken for 10 min; the mixture was centrifuged at 3000× *g* for 10 min, and the supernatant was collected. After three cycles of collection, 2-mL Eppendorf tubes were filled with the collected supernatant, and the samples were evaporated to dryness under vacuum centrifugation in a Uni trap UT-1000 evaporator (EYELA, Tokyo, Japan) at 40 ± 3 °C. Finally, 100 μL of acetonitrile was added to the tubes to mix with and dissolve the remaining SSM, and the obtained solution was analyzed through HPLC–fluorescence detection.

The measured SSM content in plasma was validated through selectivity, linearity, accuracy, and precision measurements. To confirm the selectivity of SSM, we inspected the interference that appeared at the retention time. A working standard solution of SSM (20 μL) was spiked with blank rat plasma (180 μL) to plot calibration curves over the range of 1.0–500 ng/mL. Moreover, a linear regression curve of the form y = 20389x + 104,357 (R^2^ = 0.9999) was used for fitting, and the LOQ was 1.0 ng/mL.

The HPLC–fluorescence detection system consisted of an L-7100 pump, L-7200 autosampler, and L-7485 fluorescent detector (Hitachi, Tokyo, Japan). The analytical column was a reverse-phase Luna C18 column (250 × 4.6 mm, 5 μm; Phenomenex, Torrance, CA, USA). The mobile phase comprised acetonitrile and water at a ratio of 70:30 (*v*/*v*), and its flow rate was 1 mL/min. The excitation and emission wavelengths of the fluorescent detector were 290 and 320 nm, respectively.

### 3.11. Data Analysis

Data are expressed as means ± standard deviations. Pharmacokinetic parameters were derived using a method described by a previous study [38]; specifically, parameters were calculated through a noncompartmental analysis. Group comparisons were performed using a one-way analysis of variance or paired-sample t-test, followed by a least significant difference test. All statistical analyses were performed using SPSS v14.0 (SPSS, Inc., Chicago, IL, USA). Statistical significance was set at *p* < 0.05 or 0.001.

## 4. Conclusions

In this study, we successfully developed a suitable SNEDDS dosage formulation for SSM absorption. The optimized formulation was determined to exhibit a self-emulsification ability, favorable transmittance (%), and an adequate droplet size range. The solubility test results indicate that SSM’s solubility in the F10 formulation was greater than that in Tween 80 (1%, *w*/*v*) by 160-fold. Moreover, the permeability of SSM in the intestinal segments increased by approximately 3- to 4.4-fold. SSM–SNEDDS administration engendered a significant improvement in C_max_ (9-fold) for SSM; compared with that in the SSM solution formulation, the relative bioavailability of SSM in the SSM–SNEDDS formulation increased significantly (approximately 12.9-fold). In conclusion, we present a simple method for improving the hydrophilicity, stability, and bioavailability of SSM. This may further enhance the therapeutic value of SSM.

## Figures and Tables

**Table 1:** Composition of SSM self-nanoemulsifying drug delivery system (SNEDDS) formulations and the results of the dispersibility test, droplet size, polydispersity index (PDI), and transmittance (%).

Formulation	GT/Tween20 (*w*/*w*)	Dispersibility		Droplet Size (nm)	PDI	Transmittance (%)
		Water	0.1 N HCl			
F1	1:1	E	E	N/A	N/A	N/A
F2	1:3	D	D	156.5 ± 11.3	0.27 ± 0.01	1.2
F3	1:5	C	C	166.1 ± 7.9	0.22 ± 0.05	0.1
F4	1:7	C	C	107.1 ± 4.9	0.18 ± 0.02	57.6
F5	1:9	C	C	90.7 ± 4.7	0.08 ± 0.06	70.2
	GT/Transcutol HP/Tween 20 (%, *w*/*w*/*w*)					
F6	10:10:80	B	B	158.7 ± 60.1	0.26 ± 0.04	4.1
F7	10:20:70	B	B	204.6 ± 126.5	0.23 ± 0.02	31.9
F8	10:30:60	B	B	200.6 ± 94.9	0.19 ± 0.03	11.7
F9	10:40:50	B	B	634.4 ± 130.1	0.28 ± 0.01	0.62
	GT/Cremophor EL/Tween 20 (%, *w*/*w*/*w*)					
F10	10:10:80	A	A	66.4 ± 31.4 *	0.05 ± 0.01 *	91.6
F11	10:20:70	A	A	142.9 ± 75.9	0.10 ± 0.01	92.5
F12	10:30:60	A	A	174.3 ± 36.7	0.15 ± 0.01	94.9
F13	10:40:50	A	A	242.5 ± 148.3	0.18 ± 0.01	95.7

GT is glyceryl trioctanoate, N/A: not applicable. * Significantly different compared to other groups (*p* < 0.05). Results are expressed as the mean ± standard deviation of the mean (*n* = 3 for each group).

**Table 2:** Pharmacokinetic parameters of SSM in rats.

	SSM Suspension (100 mg/kg, p.o.)	F10 (100 mg/kg, p.o.)	SSM Solution (1 mg/kg, i.v.)
C_max_ or C_0_ (ng/mL)	25.6 ± 3.9	231.2 ± 15.3 *	1336.5 ± 126.7
t _1/2_ (h)	4.7 ± 3.1	10.5 ± 2.4	1.2 ± 0.5
AUC_0__→__t_ (h·ng/mL)	131.9 ± 26.0	1697.9 ± 624.7 *	385.9 ± 109.9
Relative bioavailability (%)	N/A	1287.3	N/A
Absolute bioavailability (%)	0.3	4.4	N/A

C_max_/C_0_ is the maximum of the concentration, t _1/2_ is the half-life, AUC_0__→t_ is the area under the plasma concentration–time curve from zero (0) h to time (t), and N/A is not applicable. * Significantly different compared to the SSM suspension (100 mg/kg, p.o.) group (*p* < 0.05). Results are expressed as the mean ± standard deviation of the mean (*n* = 6 for each group).
